# How does the medicines retail sector ensure continued access to medicines during public health emergencies? Lessons from the COVID-19 pandemic in Uganda

**DOI:** 10.1080/20523211.2024.2418977

**Published:** 2025-01-28

**Authors:** Eleanor Hutchinson, Sunday Mundua, Jessica Myers, Sian E. Clarke, Kristian Schultz Hansen, Chrispus Mayora, Freddie Ssengooba, Freddy Eric Kitutu

**Affiliations:** a Department of Global Health and Development, London School of Hygiene and Tropical Medicine, London, UK; b Department of Community Health and Behavioral Sciences, Makerere University School of Public Health, Kampala, Uganda; c London School of Hygiene and Tropical Medicine, London, UK; d Department of Disease Control, London School of Hygiene and Tropical Medicine, London, UK; e The National Research Centre for the Working Environment, Copenhagen, Denmark; f Department of Health Policy Planning and Management, Makerere University, School of Public Health, Kampala, Uganda; g Department of Pharmacy, Makerere University School of Health Sciences, Kampala, Uganda; h Department of Women’s and Children’s Health, International Child Health and Migration, Uppsala University, Uppsala, Sweden

**Keywords:** Medicine sellers, medicine retail sector, drug shops, private clinics, pharmacies, COVID-19, Uganda, antimicrobials, pharmaceuticals, public health emergencies

## Abstract

**Background:**

The medicines retail sector (MRS) enables access to life-saving health commodities. Despite efforts to harness this market for public health goals, in low- and middle-income countries it is rarely incorporated into pandemic preparedness. This paper analyses the role of the MRS in the response to COVID-19 in Uganda, the extent to which it was incorporated into national planning and in the continuity of essential services.

**Methods:**

We conducted a cross-sectional study using sequential mixed methods in two purposively sampled rural districts in central Uganda. Qualitative research comprised 27 focus group discussions with drug shop vendors (DSVs), pharmacists, clinic staff and community members across two districts (*n* = 250); key informant interviews at national (*n* = 6) and district (*n* = 11) levels. Qualitative findings were used to modify a facility-based survey conducted in MRS outlets (*n* = 625). A household survey focusing on household dynamics and treatment seeking during COVID-19 was conducted in both districts (*n* = 1680).

**Results:**

At the national level, attempts were made to involve the MRS in policy and technical advice but this was not sustained. At the district level, almost no effort was made to include the MRS in the response to COVID-19. In the community, residents described their reliance on the MRS to provide medicines, especially during lockdowns. Medicine sellers subject to stringent rules on their movement during lockdown, reported some disruptions in tracer medicine stocks and an increase in prices at their suppliers. They adapted, finding new ways to purchase medicines but overall sales of medicines fell.

**Conclusions:**

The MRS is critical to the distribution of medicines in many countries. This remains the case or can be heightened during health crises. Pandemic preparedness must incorporate strategies to support medicine sellers to ensure ongoing access to commodities during public health emergencies.

## Introduction

The medicine retail sector (MRS) enables access to life-saving health commodities across the world (Haque et al., 2020; Kitutu et al., 2014; Mashuri et al., 2022; Mayora et al., 2018). In Asia and Africa, drug shop vendors (DSVs), patent medicine vendors, private clinics and pharmacies facilitate access to medicine for adults and children in both rural and urban centres (Beyeler et al., 2015; Ferdiana et al., 2021; Goodman et al., 2007; Wafula et al., 2012). In many places, numerous medicines retail outlets cluster around public clinics and hospitals, creating a shadow health system that responds to the limitations of underfunded public systems and the exclusions of expensive private hospitals (Hutchinson et al., 2023). In these shadow systems, informal practices often compete with and sometimes dominate the laws and rules created to govern their action (Hutchinson et al., 2023).

In recognition of its critical role, there have been almost 30 years of advocacy calling for the inclusion of the MRS in health sector planning in Africa (Adome et al., 1996) and for strategies to harness its activities for public health goals (Gelband et al., 2004). Yet, appreciation of the role that the MRS plays and its incorporation into central health planning remains limited. In Uganda, the subject of this paper, there are 1871 registered retail pharmacies, and 11,372 licenced drug shops distributed across Uganda currently registered to the National Drug Authority records (National Drug Authority, 2024) and there are 4510 private clinics registered with the Uganda Medical Dental Practitioners’ Council (Uganda Medical Dental Practitioners’ Council, 2024). In addition, there are several other drug shops and private clinics that are neither registered nor licenced and the authors are not able to establish how many there are. In these shadow systems, informal practices often compete with and sometimes dominate the laws and rules created to govern their action (Hutchinson et al., 2023). Concerns about frequent rule breaking, the highly fragmented nature of the sector and limited collective representation keep the sector at the peripheries of health system planning, programming and intervention (Hutchinson et al., 2022, 2023). This is despite examples of successful attempts to work with the MRS in Uganda to increase access to public health interventions and improve practices (Awor et al., 2014; Kitutu et al., 2014; Mbonye et al., 2013)

During public health emergencies in Africa, as public health systems respond to outbreaks and pandemic disease, ‘close to community’ providers including the MRS become increasingly important for the continuity of care and access to medicines (Iwuoha et al., 2021; Kabwama et al., 2022; Martineau, 2016). The MRS is an important supplier of medicines close-to-home when travel was restricted and government health facilities were overwhelmed with patient numbers. These were primarily for the relief of symptoms such as sore throats, headache and fevers. Once they were allowed to admit COVID-19 patients, private clinics were an important provider of in-patient care.

In this paper, we interrogate how the position of the MRS as critical service providers but peripheral policy actors shapes their ability to provide services during public health emergencies. It focuses on Uganda, where the MRS comprises drug shops, small private clinics and pharmacies (Mayora et al., 2018; Mbonye et al., 2013). Drug shops are the most numerous providers. They are increasingly occupied by professional health workers (nurses, midwives and occasionally clinical officers) unable to find work elsewhere, but most are not registered with the national drug authority (Hutchinson et al., 2022; Mayora et al., 2018; Mbonye et al., 2016). By law, they cannot sell antibiotics, but a recent study demonstrates that the majority sell these medicines and that antibiotics sales are critical to most businesses (Hutchinson et al., 2023). Ugandan pharmacies are much more likely to be licenced than drug shops, they are also reported to offer prescription-only medication to patients and care-seekers who present without valid prescriptions (Olamijuwon et al., 2022). Some drug shops operate informally as unregistered clinics with in-patient services and overnight care. Registered clinics provide inpatient care but also sell medicines including antibiotics.

The analysis draws on mixed methods research undertaken among national policymakers and in two purposively sampled districts which was conducted during the COVID-19 pandemic. It explores the involvement of pharmacies, small private clinics and drug shop representatives in the technical support organised by the Ministry of Health, their exclusion from district-level response, and the ways in which members of the MRS organised themselves to ensure continuity of supply medicines to community members especially during strict lockdowns and curfews.

## Methods and materials

### Study design

This was a sequential convergent mixed methods study where Focus Group Discussions (FGDs) with purposively sampled MRS providers and community members preceded quantitative surveys. It aimed to explore how the public health response to COVID-19 shaped the supply of and access to treatment in the medicines retail sector in Uganda; to describe how policy can be adapted to ensure continuity of access to essential medicines and support the involvement of the retail sector in COVID-19 activities during this and future outbreaks. Key Informant Interviews (KIIs) were also conducted with the Ministry and district officials. Qualitative observations informed the quantitative components to enable corroboration and triangulation of the effect of the COVID-19 pandemic and the role played by the MRS.

### Study setting

**Figure 1.**
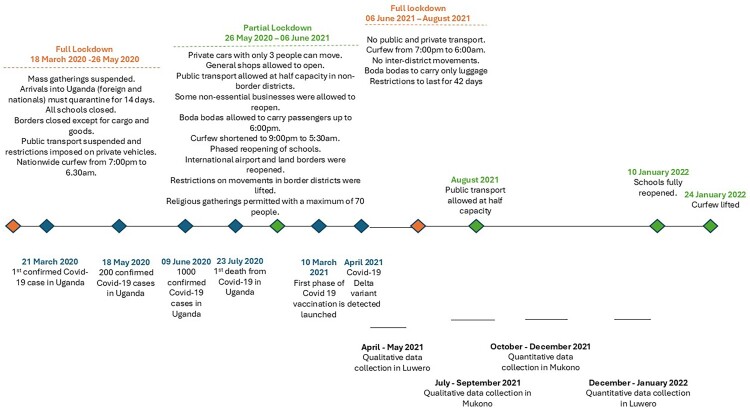
Timeline of the government’s response to COVID-19 and the gathering of data in the districts for the present study.

The Ugandan government responded to the emerging pandemic by establishing a command-and-control approach (Khisa & Rwengabo, 2023; Parker et al., 2022). At the national level, technical guidance was provided by the Ministry of Health to a National Level Task Force under 8 key pillars. At the district level, multi-sector district task forces were created to coordinate case management, surveillance, health promotion, resource mobilisation, enforcement of control measures and continued delivery of basic services. These were chaired by the most senior political appointee at this level, the Resident District Commissioner (RDC) (Mbabazi & Kasalirwe, 2020). The RDCs oversaw the first lockdown and the following 21 months when restrictions were put in place to respond to the waves of the disease (see Figure 1). Supported by paramilitary local defense units (LDUs) and the police, the Uganda People’s Defense Force (UPDF) was tasked with ensuring that people lived by the national standard operating procedures (SOPs).

Shortly before the second lockdown (in 2021), the study began in two purposively sampled districts – Luwero and Mukono. Both districts are predominantly rural with agrarian economies. As Table 1 shows, both are served by large numbers of private providers. The overwhelming majority of health facilities in both districts provide primary care only (Ministry of Health, 2018).

**Table 1.** Population and number of health facilities in the study setting.

	Luwero	Mukono
Population estimate (Uganda Bureau of Statistics, 2022)	535,000	701,400
Government health facilities (Ministry of Health, 2018)	41	40
Private not for profit facilities (Ministry of Health, 2018)	26	22
Private for-profit clinics (Ministry of Health, 2018)	18	51
Registered drug shops (National Drug Authority, 2023a)	212	341
Registered pharmacies (National Drug Authority, 2023b)	59	121

### Study population, selection, and sampling strategy

Enumerators trained in the study protocol, procedures and research ethics mapped all the drug shops, private clinics, and pharmacies in the Luwero and Mukono districts. Villages within their catchment areas were mapped for the community FGDs and household survey. Qualitative research comprised purposively sampled interviews and focus group discussions (FGDs). FGD participants from MRS were selected purposively as follows and separated between MRS group (drug shops, pharmacies, clinics), rural or urban, and those in operation for 5 years or more.

FGDs with the MRS and community explored experiences of COVID-19 including the public health restrictions that had been put in place to mitigate its impact. KIIs explored the extent to which, how and why members of the MRS had been involved in district or national-level activities.

KII interviewees were involved in district or national-level task forces. FGD participants were identified from lists provided by the National Drug Authority, the District Health Office, the National Drug shop Advocacy Initiative (drug shops only) and Allied Health (clinics only). All clinics and pharmacies were registered. Registered and unregistered drug shops were included. Village Health Team members assisted study enumerators in mobilizing FGD participants for the community FGDs. They were purposively sampled to include men, women and caretakers of children under five.

Findings from the qualitative data were presented in weekly meetings with team members and these were used to adapt the quantitative tools used in a previous study on the MRS.

We included a representative sample of MRS providers and community members in each district for the quantitative survey. A structured questionnaire was conducted in 625 medicine outlets across both districts. The sample size was determined to assess differences among drug shops, private clinics and pharmacies in the two study districts of Luwero and Mukono, and as such the final sample size incorporated probability proportional to size to account for the variation in the number of different medicine outlets in the sampling frame. The community survey was conducted in 84 villages – taking a village as a cluster – across the two districts and included 20 households per village chosen using systematic random sampling. One adult per household who had slept in the house for at least 4 weeks preceding the survey was interviewed.

### Data collection strategy, tools, and procedure

The MRS, KII and FGD questions focused on changes made to the business; training and intervention by government or non-governmental organisations; desire to be involved in public health plans, testing, and treating patients. FGDs with community members focused on experiences of health care during the pandemic, access to and availability of medicines. KIIs sought to understand how and why members of the MRS had been involved in or excluded from the district and national-level activities.

FGD participants were approached in person in their shops or clinics or on their mobile phones. KII participants were approached on their mobile phones. The interviewer described the project, provided an information sheet and invited them to participate. FGDs were held in a local church hall. KIIs were held at the participants’ offices. Researchers read out the information sheet and asked participants to consent by signing the form provided prior to the interview/FGD. All qualitative data were recorded onto digital recorders.

Trained interviewers gained consent from those who had worked in the selected business for at least three months and who were over the age of 18 years. Only medicine outlets that had been in existence of six months or more at the survey date were eligible for the study. The enumerators had qualifications of bachelor’s or master’s level training in social or health sciences, Data were collected using electronic tools based on the KoboToolbox platform (Harvard Humanitarian Initiative. KoBoToolbox. 14 Story St, Cambridge, MA 02138) installed on secure tablets and transmitted to a secure server under the direct supervision of the principal investigators. Data were gathered on background characteristics of medicine outlets and drug shop attendants, medicines and stocks affected by the occurrence of the COVID-19 pandemic including any increases in prices.

### Data management and analysis

We conceptualised the MRS as a social field that emerges and changes as differently positioned individuals to compete and cooperate over scarce (social, economic, medical, health related) resources (Hutchinson et al., 2022). Qualitative data were transcribed, translated and uploaded into Nvivo V.12. Two researchers (JM and SM) used a combination of deductive and inductive coding under guidance from EH to explore experiences among different providers. Quantitative data from MRS and household surveys were analysed as follows; numerical variables were summarized as means with standard deviation and categorical variables were summarised as proportions and frequencies and presented in a table and associated assessed by the Chi-square test. Additional details of the quantitative data collection have been published elsewhere (AUTHOR paper under review).

No patients or members of the public were involved in the design of the study.

#### Reflexivity statement

All of the researchers collecting data were Ugandan. Two of the qualitative researchers were experienced in undertaking participant observation, in-depth interviews and FGDs in drug shops. One was a pharmacist. Researchers were trained to consider the ways in which they might be identified as working with the government and that their own biases, especially about what constitutes ‘good practice’ might impact on the data collected. Findings were triangulated between IDIs and the three groups that made up the FGDs.

### Ethics

The research was approved by the Ugandan National Council for Science and Technology (reference number HS1302ES) Makerere University School of Health Sciences REC (reference number MAKSHSREC-2020-71) and London School of Hygiene and Tropical Medicine Ethics Committee (reference number 22907). COVID-19 public health measures including using alcohol rub, wearing face masks and social distancing were observed to prevent transmission and protect the research team and study respondents.

## Results

### Background characteristics

Twenty-seven focus group discussions were conducted with DSVs clinic staff and pharmacy staff in the Luwero and Mukono districts. Twelve FGDs were completed with household participants (128). The minimum number of participants in each FGD was 5, and the maximum was 12 (see Table 2 for details). Seventeen KIIs were completed with health officials at the district (11) and national level (6).

**Table 2.** Participants in the focus group discussions.

		Number of FGDs	Total number of participants
DSV	Luwero	4	34
	Mukono	3	31
Clinic staff	Luwero	2	14
	Mukono	4	31
Pharmacy staff	Luwero	1	5
	Mukono	1	7
Community	Luwero	6	64
	Mukono	6	64
Total		27	250

In the community, we surveyed a total of 1680 participants (860 from Luwero and 820 from Mukono). Most participants (76%) were women, and the average age was 41 years. In the MRS, 625 outlets were surveyed (263 in Luwero, 362 in Mukono). The majority of these were drug shops (53%), followed by clinics (36%) and pharmacies (11%). As Table 3 shows, drug shops and clinics were the least likely to be licenced (approximately 30% were unlicenced in these groups). Almost all the pharmacies were licenced (98%). Overall, 65% of the medicines’ sellers were women. This was unevenly distributed. In drug shops, women represented 80% of those interviewed, whereas in private clinics there were only 46% of staff. Most of the medicine sellers in the districts were comprehensive nurses (67%). Most of the outlets employed one or two staff members (66%) and were stand-alone enterprises (63%) rather than being a chain or one of many outlets under the same ownership.

**Table 3.** Background characteristics of the medicine outlets in the sample.

Characteristic	Total (%)	Number of medicine outlets (%)			*p* value
		Drug shops (%)	Private clinics (%)	Pharmacy (%)	
Years of existence					
Under 1 year	64 (10.3)	30 (9.0)	25 (11.3)	9 (12.9)	0.240
1–3 years	256 (41.0)	132 (39.6)	98 (44.3)	26 (37.1)	
4–6 years	101 (16.2)	48 (14.4)	42 (19.0)	11 (15.7)	
More than 6 years	120 (19.2)	71 (21.3)	36 (16.3)	13 (18.6)	
No response	83 (13.3)	52 (15.6)	20 (9.1)	11 (15.7)	
Number of staff working at medicine outlet					
One	203 (32.5)	177 (53.1)	24 (10.9)	2 (2.9)	<0.0001
Two	208 (33.3)	139 (41.7)	52 (23.5)	17 (24.3)	
Three	81 (13.0)	12 (3.6)	47 (21.3)	22 (31.4)	
More than three	132 (21.2)	5 (1.5)	98 (44.3)	29 (41.4)	
Licence status for current year					
Has licence for current year	386 (61.8)	188 (56.5)	134 (60.6)	64 (91.4)	<0.0001
Applied but not out	103 (16.5)	67 (20.1)	32 (14.5)	4 (5.7)	
Does not have	123 (19.7)	71 (21.3)	50 (22.6)	2 (2.9)	
Does not know	12 (1.9)	7 (2.1)	5 (2.3)	0	
Licence status for previous year (*N* = 477)					
Has licence for previous year	352 (73.8)	179 (71.3)	124 (70.5)	49 (98.0)	<0.0001
Applied but not out	94 (19.7)	60 (23.9)	33 (18.8)	1 (2.0)	
Does not have licence for previous year	20 (4.2)	9 (3.6)	11 (6.3)	0 (00.0)	
Does not know	11 (2.3)	3 (1.2)	8 (4.6)	0 (0.0)	
Number of outlets under same ownership					
One	392 (62.8)	228 (68.5)	136 (61.5)	28 (40.0)	<0.0001
Two	167 (26.8)	74 (22.2)	60 (27.2)	33 (47.1)	
Three	10 (1.6)	5 (1.5)	3 (1.4)	2 (2.9)	
More than three	6 (0.96)	5 (1.5)	0 (0.0)	1 (1.4)	
Does not know or refused to answer	49 (7.9)	21 (6.3)	22 (10.0)	6 (8.6)	

Except for years of existence, there were differences among the drug shops, private clinics and pharmacies regarding number of staff working at the outlet (*p* value <0.0001), licencing status for current year (*p* value <0.0001), licencing status for previous year (*p* value <0.0001) and number of outlets under the same ownership (*p* value <0.0001). The authors also sought to understand and characterize the drug shops, private clinics and pharmacies that constitute the medicine retail sector in the context of Uganda and to demonstrate that although these outlets operate in the same health market ecosystem, often targeting the same clients and customers, they are structurally different. The authors intended to demonstrate the inherent differences as shown by statistically significant differences and that these differences have implications for how interventions to shape them and improve their functioning should be constructed. The authors seek to differ from other researchers who have previously approached these different categories of medicine retail outlets as homogenous. Our study shows that they are not the same, in fact they are heterogeneous and should be approached as such.

### Policy and interventions at national and district level

At the beginning of the COVID-19 pandemic, it appeared that the MRS would be involved in the national response. Researchers advising the National Task Force raised the importance of private sector provision of care and medicines in the country (National KII #1, 17/11/2022). Representatives for private sector providers (National Drug Shop Advocacy Initiative, the Pharmaceutical Society of Uganda and the Uganda Health Care Federation who represent private clinics) were then invited to technical meetings within the Ministry of Health from March 2020 (KII #1 25/01/2023, KII #4 25/01/2023, KII #6 15/02/2023).

In April 2020, when the plan for continuity of essential services was published, the MRS was identified as a critical partner for malaria treatment and family planning. Resident District Commissioners were urged to involve the sector in local planning and response. The National Task Force advised that MRS staff should be exempt from travel bans and able to move freely and safely. This initial enthusiasm, however, gave way to reluctance and confusion around how the MRS could be involved in the response and what it could be asked to do. When the National Strategy was finally published, no mention was made of the MRS. Policymakers reported finding it increasingly difficult to involve the MRS in national planning as the pandemic unfolded (National KII #4, 20/01/2023).

Among Ministry of Health officials, powerful concerns were raised against the private sector (including drug shops, small private clinics and pharmacies), who were seen as untrustworthy partners who were using the crisis to increase their profits (KII #4, 25/01/2023). Policymakers and officials who remained supportive of the MRS became frustrated with the lack of coordination among and between private sector providers, a factor that undermined any attempt to foster collaboration between them and the state.

Despite this, national level policymakers described how the decision to allow medicine sellers to travel had been communicated at the district level (for example, ‘the Resident District Commissioners accepted that when they found a health worker going to a drug shop, they should not beat them’, KII #1 25/01/2023). At the district level, however, we found no evidence of action to include the MRS in the response or the alteration of the lockdown rules for the MRS. Neither district had involved drug shops or pharmacies in their responses nor did they have a plan for how this might be achieved. Few clinic staff, and no pharmacists or drug shop vendors (DSVs) had received any personal protective equipment from the district officials. No action was taken to ensure that medicine sellers could travel to stock their shops and although some clinic staff were provided with training from the district health team, drug shops and pharmacies were not. As Table 4 sets out, providers in the MRS in both districts were subject to severe restrictions on their movement and ability to provide care for their clients.

**Table 4.** Restrictions to the MRS during the first lockdown**.**

	Subject to closures/curfew	Travel restrictions	Knowledge training on COVID-19
Drug shops	Allowed to open until 6pm	Full restrictions on movement unless staff have an up-to-date health worker ID	No formal training in Mukono or Luwero
Pharmacies	Allowed to open until 6pm	Full restrictions on movement unless staff have an up-to-date health worker ID	No formal training in either district
Clinics	Able to remain open at night but staff must stay at the facility or very nearby.	Full restrictions on movement unless staff have an up-to-date health worker ID	Some formal training of clinic staff in larger private clinics in both districts.

### Accessing care in the community

In the community, rural inhabitants described having relatively few problems in accessing care when the first lockdown began. Community health workers (CHW) provided treatment for respiratory infections, malaria and diarrhoea, and drug shops still had stock and remained open during the day (for example, ‘We had the Village Health Teams or [i.e. CHWs] and they would give us tablets and if it failed you would take the children to a drug shop/clinic nearby’, FGD Women caring for children <5 Luwero).

As the first lockdown progressed, however, CHWs ran out of supplies. Permits to travel, including for healthcare, had to be provided by local officials but it was often hard for them to find the relevant authority. In both districts, but particularly Mukono, fear of military and police violence could prevent them from going to the health facilities at all (‘Even if you had a travel permit they would beat you first before you present it..even those that had it they would get beaten’ – FGD14-RuralWomen-Luwero-27.05.21). Those seeking emergency care who relied on public services during the lockdowns were particularly vulnerable. Pregnant women were transported by bicycle through country paths by bicycle to avoid the military roadblocks and a lack of transport had devastating effects. As one woman described:

COVID-19 affected me so much. I was pregnant, I gave birth here at (lower-level health centre) but the child became ill. We called the ambulance from (district hospital) to come and pick us but they wanted us to send them money first yet the child was badly off, we sent the money but the ambulance had a mechanical problem. We got a boda-boda, but the child died on the way.

For those who managed to get to public health facilities, stockouts were more difficult to manage than usual; long waiting times left too few hours to travel to drug shops to buy pharmaceuticals and return home before curfew. With difficulties in accessing care from the public sector, FGD responses suggest that the MRS maintained its position as an important point of access for medicines and advice.

### Challenges providing information and care in the MRS

Staff working in the MRS, especially those who lived away from their facilities, found that the first lockdown was very difficult. Officially, those with health worker identity cards were allowed to move freely at this time, but many did not have these documents (see below for discussion). If an identity card expired, it was impossible to go to the respective council to renew it. Providers without relevant IDs reported moving about at night on bicycles with no lights to avoid detection, but even those with uniforms and relevant documentation reported that would stop them frequently to check IDs and demand bribes. The beginning of the first lockdown also saw the police and military attacking care providers. (For example, ‘the police didn’t know that the drug shops could continue working so they could even beat us. One time they came and banged the door when I had a client there who had been involved in an accident’ FGD 1-unlicenced drug shop-Luwero-27.04.21.)

These travel restrictions also made it very difficult for DSV and clinic staff to restock medication. Usually, DSVs shop around to get the cheapest prices and with little capital, they re-stock their shops frequently with small amounts of medicine. During the lockdown, DSVs and clinic staff resorted to using third parties to make purchases – sending motorcycle taxis (boda-boda) with the lists of medicines to wholesale pharmacies in the districts. This enabled some medicines to flow into shops and clinics, but DSVs who barter with pharmacy staff over the prices of medicines were unable to make on-the-spot decisions about the amounts to pay and which medicines to buy when prices rose, or stock was unavailable. Boda-boda drivers often purchased the wrong stock, paid excessively high prices and sometimes failed to bring back medicines at all.

I sent a boda-boda rider with a list of drugs that I wanted. The pharmacy gave him other medicines. It was a lot of medicine. I was furious, I had spent the only money I had and I didn’t have any more money. I called them and they said, “We didn’t have those drugs that you wanted, the next time he is coming back this side, let him bring those drugs back.” I said, “Now what is the use of this medicine to me, and if I give it to the boda-boda rider to bring it back to you it will cost me more money. The boda-boda rider will make money as I make a loss.” – FGD5-DrugShop-Luwero-29.04.21

Boda-boda drivers doubled the cost of transporting medicines during lockdowns. The prices of medicines increased while clients many of whom were unable to work at this time were increasingly requesting a deferment of payment, credit and half doses of medicines to cut costs.

Despite these measures, DSVs and clinic staff often found it difficult to stock their shops. Medicine sellers reported stockouts at their suppliers and an increase in the prices at which they bought their stock during the FGDs.

As Table 5 shows, two-thirds of the medicine outlets reported that their suppliers stocked out contraceptives, more than a third stocked out antimicrobials, about a fifth stocked out on antimalarials, face masks and other PPE, and less than a fifth stocked out on alcohol rub. The extent of stock out varied among drug shops, private clinics, and pharmacies for antimicrobials (*p* value, 0.021) and alcohol handrub (*p* value, 0.039)

**Table 5.** Percentage of medicine outlets that reported stock outs of tracer health commodities at their suppliers in the period from February 2020**.**

Variable	Combined *N* = 624 (%)	Category of medicine outlet			*P* value
		Drug shops *N* = 333 (%)	Pharmacies *N* = 70 (%)	Private clinics *N* = 221 (%)	
Contraceptives					
Suppliers stocked out	367 (58.8)	201 (60.4)	47 (67.1)	119 (53.9)	0.159
Suppliers not stocked out	231 (37.0)	121 (36.3)	19 (27.1)	91 (41.2)	
Do not know	26 (4.2)	11 (3.3)	4 (5.7)	11 (5.0)	
Antimicrobials					
Suppliers stocked out	238 (38.1)	118 (35.4)	36 (51.4)	84 (38.0)	0.021
Did not	373 (59.8)	204 (61.3)	33 (47.1)	136 (61.5)	
Do not know	13 (2.1)	11 (3.3)	1 (1.4)	1 (0.5)	
Antimalarials					
Suppliers stocked out	132 (21.2)	76 (22.8)	19 (27.1)	37 (16.7)	0.189
Suppliers did not	484 (77.6)	254 (76.3)	50 (71.4)	180 (81.5)	
Do not know	8 (1.3)	3 (0.9)	1 (1.4)	4 (1.8)	
Face masks or other personal protective equipment (PPE)					
Suppliers stocked out	132 (21.2)	56 (16.8)	17 (24.3)	59 (26.7)	0.060
Suppliers did not	483 (77.4)	272 (81.7)	52 (74.3)	159 (72.0)	
Do not know	9 (1.4)	5 (1.5)	1 (1.4)	3 (1.4)	
Alcohol handrub					
Suppliers stocked out	108 (17.3)	64 (19.2)	14 (20.0)	30 (13.6)	0.039
Suppliers did not	503 (80.6)	263 (79.0)	55 (78.6)	185 (83.7)	
Do not know	13 (2.1)	6 (1.8)	1 (1.4)	6 (2.7)	

They also increased the prices at which they sold medicines in their shops and clinics and swapped medicines. Rumours about different treatments that could be used in the management of COVID-19 also created surges in demand for certain medicines including chloroquine and Vitamin C tablets.

Table 6 shows how retail outlets reported that suppliers increased the cost of tracer health commodities, with antimicrobials (84.9% of medicine outlets) the most affected, followed by alcohol handrub (76.1%), face masks and PPE (70.5%), contraceptives (59.3%) and lastly antimalarials (51.8% of the medicine outlets). However, there were differences in the proportions of drug shops, private clinics and pharmacies affected by supplier increase of prices.

**Table 6.** of medicine outlets sampled that reported that their suppliers increased prices of tracer health commodities during the period from February 2020**.**

Health commodity	Combined *N* = 624 (%)	Category of medicine outlet			*P* value
		Drug shops *N* = 333 (%)	Private clinics *N* = 221 (%)	Pharmacies *N* = 70 (%)	
Contraceptives					
Suppliers increased prices	370 (59.3)	217 (65.2)	33 (47.1)	120 (54.3)	0.011
Suppliers did not	213 (34.1)	94 (28.2)	33 (47.1)	86 (38.9)	
Do not know	41 (6.6)	22 (6.6)	4 (5.7)	15 (6.8)	
Antimicrobials					
Suppliers increased prices	530 (84.9)	280 (84.1)	65 (92.9)	185 (83.7)	0.021
Suppliers did not	80 (12.8)	41 (12.3)	4 (5.7)	35 (15.8)	
Do not know	14 (2.2)	12 (3.6)	1 (1.4)	1 (0.5)	
Antimalarials					
Suppliers increased prices	323 (51.8)	198 (59.5)	30 (42.9)	95 (43.0)	0.001
Suppliers did not	290 (46.5)	130 (39.0)	38 (54.3)	122 (55.2)	
Do not know	11 (1.8)	5 (1.5)	2 (2.9)	4 (1.8)	
Face masks or other personal protective equipment					
Suppliers increased prices	440 (70.5)	207 (62.2)	53 (75.7)	180 (81.5)	<0.001
No	169 (27.1)	115 (34.5)	16 (22.9)	38 (17.2)	
Do not know	15 (2.4)	11 (3.3)	1 (1.4)	3 (1.4)	
Alcohol handrub					
Suppliers increased prices	475 (76.1)	238 (71.5)	57 (81.4)	180 (81.5)	0.045
Suppliers did not	122 (19.6)	75 (22.5)	11 (15.7)	36 (16.3)	
Do not know	27 (4.3)	20 (6.0)	2 (3.0)	5 (2.3)	

### Responding to lockdowns and changing practice in the MRS

Despite these challenges, very few shops, clinics or pharmacies reported closing their businesses. Many workers lived close by, so did not have to travel, nurses working in some larger private clinics in the districts were given overnight accommodation.

In urban areas, DSVs with identification documents walked into town or borrowed bicycles to go and purchase medicines. Some also hid in the back of cars or made payments to officials at roadblocks to get to neighbouring districts where they could purchase drugs more cheaply (for example, ‘Those of us who obtain drugs from Kayunga had to insert in a lot of money. When you want to bypass the security people, at each point you pay something’ – FGD2-DrugShop-Mukono-18.08.21).

To reduce costs, a small group of DSVs in Mukono pooled their money to pay taxis to collect medicines from the district wholesale pharmacies to distribute them. Later, pharmacies started to take orders over the phone and deliver medicines to drug shops, hiring boda-bodas or using their own cars or vans. In Luwero, however, it was difficult to get out to the deepest rural areas and back to the pharmacy before curfew, limiting the shops that they could deliver medicines to.

For the customers, we had boda-bodas but time was not enough. The boda riders who delivered the medicine had to have money to bribe the police officers so that they may move smoothly … . we would give him some small money for such cases and he would give the officers something small. – FGD7-Pharmacy-Luwero-10.05.21

Clinic staff, DSVs and pharmacists were keen to exploit new markets for medicines created by the pandemic and rumours about treatments. They were, however, often unable to get access to these medicines. Staff in clinics in both districts raised their prices of IV medicines, which have long been seen as a more powerful form of care delivery in Uganda (Birungi, 1998). This was undoubtedly a means of making additional funds but also, they argued, a way of protecting supplies that were needed for critically ill patients. In contrast to discourses of profiteering across private sector providers in much of the Ugandan media, our data suggest that overall sales of medicines in pharmacies, clinics and drug shops fell dramatically from March to the end of May 2020 and the proportion of outlets that made a financial loss rose from 14% to 22%.

## Discussion

During health emergencies, public health systems take control of mitigation and treatment of patients by default, and communities commonly cope by seeking healthcare from close to community health providers (Laverack & Manoncourt, 2016; Martineau, 2016). Evidence is emerging that this was the case during the COVID-19 pandemic and that their importance was compounded by restrictions on movement (Iwuoha et al., 2021). At the global level, as part of their ‘whole of government, whole of society’ approach, the WHO advised governments to leverage the private sector and ensure that they were aligned with national-level responses (Clarke et al., 2020; World Health Organization, 2021). Our findings suggest that in Uganda, the lack of involvement of the MRS in the pandemic response comes from an ongoing failure at the national and district levels to recognise the important benefits of leveraging MRS for essential health service continuity. While some policymakers were aware of the need to engage the MRS widespread concerns about profiteering during a crisis undermine policymakers commitment to this involvement. Yet, vital processes through which the MRS could have been supported to sustain stocks and supply, stabilise health commodity prices, and therefore better support community resilience to epidemics and outbreaks were missing.

In our study, DSVs and clinic staff complained that they lacked knowledge about COVID-19. Work by Olamijuwon et al. (2022), suggests that in comparison to their Tanzanian counterparts (working in the Tanzanian equivalent of a drug shop), Ugandan DSVs were better able to provide advice about how to identify and manage COVID-19 and were more concerned with implementing social distancing measures (Olamijuwon et al., 2022). This could have been related to the fact that the Tanzanian government’s response to COVID-19 was marked by denial of the significance of the virus to the population and their information campaign was much less intensive in approach in comparison to the national response in Uganda.

Going forward, this sector would benefit from better more effective representation that could ensure that policy decisions are understood and acted upon at the district level. Our research shows that even when policy is created to support the MRS, it may not be effectively transferred into the districts nor translated into action at the meso or micro-level. Researchers interested in the MRS and the role that it could play in future pandemics have proposed that capacity-building initiatives (mentoring, coaching and supervision) could strengthen the relationship between public and private providers (Kabwama et al., 2022). Previous research by this team and others have shown that within the MRS the formation of representative organisations of health providers working in the MRS are becoming more commonplace. It could be that these organisations could help to make initiatives more sustainable and locally relevant (Chukwuocha et al., 2021; Hutchinson et al., 2022, 2023).

This study had several limitations. Our findings were limited to two districts and are unlikely to reflect all the experiences across districts in Uganda. In Lamwo district, for example, health workers in private clinics received training on services for pregnant women and malaria and pneumonia treatment as part of the response to COVID-19 (Kabwama et al., 2022). Although we trained qualitative fieldworkers to stress the confidentiality of the research, members of the MRS could have been concerned about discussing informal practices in these more formal settings.

## Conclusion

During the COVID-19 pandemic, public health services and personnel were given over to managing COVID-19 patients. Access to public clinics and hospitals became more difficult. The MRS had a clear role to play in halting transmission and sustaining access to treatment and care for non-COVID-19 illnesses in many countries. Despite good knowledge of the role that the MRS plays in the Ugandan health system and an interest in involving them in the national level response, at the district level little was done to support their ability to continue to provide care during the pandemic for COVID-19. In countries like Uganda where the MRS is an important provider of care and medicines, pandemic preparedness policy could find more effective ways to incorporate these actors and ensure that they are able to continue to supply access to life-saving medicines.

## Data Availability

The data sets used and/or analysed during the current study are available from the corresponding author upon reasonable request.
